# Supplementary material for: Neurological Prognostic Factors in Hospitalized Patients with COVID-19
Source: Brain Sci. 2022 Jan 30;12(2):193. doi: 10.3390/brainsci12020193 (PMC8870483; doi:10.3390/brainsci12020193)
Supplement: Supplementary file 1 [file brainsci-12-00193-s001.zip › brainsci-1506957-supplementary.pdf]

Table S1. Patients' characteristics according to the severity of neurological symptoms and signs (NSS)

		High-risk NSS			Low-risk NSS			High-risk / or absence of low-risk NSS		
	All patients n=349	No n=272	Yes n=77	P-value	No n= 103	Yes n=246	P-value	No n= 210	Yes n=117	P-value
Demographics										
Age (years)	64 (51-77)	61 (49-71)	76 (66-84)	<0.001	70 (57-79)	61 (49-73)	0.008	60 (49-69)	71 (57-80)	<0.001
Age >65 years, n (%)	167 (47.85)	102 (38.06)	62 (80.52)	<0.001	47 (57.32)	102 (41.46)	0.012	74 (32.54)	74 (63.25)	<0.001
Female sex, n (%)	191 (54.72)	150 (55.97)	39 (50.65)	0.408	41 (50.00)	141 (57.32)	0.248	119 (56.67)	62 (52.99)	0.521
Comorbidities and treatment										
Hypertension, n (%)	209 (59.89)	148 (55.22)	58 (73.52)	0.002	57 (69.51)	137 (55.69)	0.027	112 (53.33)	81 (69.23)	0.005
Obesity, n (%)	65 (18.62)	51 (19.03)	14 (18.18)	0.866	13 (15.85)	50 (20.33)	0.373	42 (20.00)	21 (17.95)	0.652
Smoking, n (%)	54 (15.47)	38 (14.23)	15 (19.48)	0.261	16 (19.51)	32 (13.01)	0.149	29 (13.81)	19 (16.24)	0.551
Diabetes mellitus, n (%)	90 (25.79)	59 (22.01)	31 (40.26)	0.001	25 (30.49)	57 (23.17)	0.185	42 (20.00)	40 (34.19)	0.005
Ischemic heart disease, n (%)	59 (16.91)	36 (13.43)	22 (28.57)	0.002	19 (23.17)	36 (14.63)	0.073	25 (11.90)	39 (25.64)	0.002
Prior CNS disease. n (%)	77 (22.06)	56 (17.72)	21 (63.64)	<0.001	25 (30.49)	33 (13.41)	<0.001	21 (10.00)	36 (30.77)	<0.001
Asthma / COPD, n (%)	20 (5.73)	15 (5.60)	3 (3.90)	0.554	1 (1.22)	18 (7.32)	0.041	14 (6.67)	4 (3.42)	0.312
Neoplasm, n (%)	40 (11.46)	31 (11.57)	9 (11.69)	0.976	12 (14.63)	25 (10.16)	0.268	23 (10.95)	14 (11.97)	0.782
Chronic kidney disease stage 3, n (%)	17 (4.87)	14 (5.22)	3 (3.90)	0.635	3 (3.66)	13 (5.28)	0.553	11 (5.24)	5 (4.27)	0.698
Immunosupressive treatment, n (%)	18 (5.16)	14 (5.22)	4 (5.19)	0.991	3 (3.66)	14 (5.69)	0.472	12 (5.71)	5 (4.27)	0.573
First COVID-19 symptoms										
Fever, n (%)	207 (59.31)	167 (62.31)	39 (50.65)	0.066	40 (48.78)	155 (63.01)	0.024	139 (66.19)	56 (47.86)	0.001

Cough, n (%)	219 (62.75)	177 (66.04)	42 (54.55)	0.065	45 (54.88)	165 (67.07)	0.047	146 (69.52)	64 (54.70)	0.008
Sore throat, n (%)	44 (12.61)	40 (13.93)	4 (5.19)	0.024	5 (6.10)	37 (15.04)	0.036	35 (16.67)	7 (5.98)	0.006
Loss of appetite, n (%)	105 (30.09)	97 (27.79)	6 (7.80)	<0.001	9 (10.98)	95 (38.62)	<0.001	91 (43.33)	12 (10.26)	<0.001
Dyspnea, n (%)	178 (51.00)	132 (49.25)	44 (57.14)	0.222	29 (35.37)	133 (54.07)	0.034	111 (52.86)	51 (43.59)	0.108
Abdominal pain, n (%)	67 (19.20)	55 (20.52)	11 (14.29)	0.220	7 (8.54)	58 (23.58)	0.003	51 (24.29)	13 (11.11)	0.004
Neurological symptoms and signs										
Headache, n (%)	130 (37.24)	117 (44.15)	13 (20.63)	<0.001	0 (0.00)	130 (52.85)	<0.001	117 (55.71)	13 (11.11)	<0.001
Dizziness, n (%)	78 (22.35)	78 (24.91)	12 (19.05)	0.326	0 (0.00)	78 (31.71)	<0.001	66 (31.43)	12 (10.26)	<0.001
Decreased mood, n (%)	44 (41.26)	122 (46.04)	21 (33.87)	0.082	0 (0.00)	144 (58.54)	<0.001	122 (58.10)	21 (17.95)	<0.001
Memory or concetration difficulties, n (%)	57 (16.33)	35 (13.21)	21 (33.33)	<0.001	9 (10.98)	47 (19.11)	0.901	31 (14.76)	24 (20.51)	0.183
Fatigue, n (%)	200 (57.31)	175 (66.04)	24 (38.71)	<0.001	0 (0.00)	200 (81.30)	<0.001	75 (83.33)	24 (20.51)	<0.001
Visual disturbances, n (%)	26 (7.45)	22 (8.30)	4 (6.45)	0.678	4 (4.88)	22 (8.94)	0.238	19 (9.05)	7 (5.98)	0.326
Decreased level of consciousness, n (%)	57 (16.33)	0 (0.00)	56 (72.73)	<0.001	19 (23.17)	23 (9.39)	0.012	0 (0.00)	42 (35.90)	<0.001
Delirium, n (%)	24 (6.88)	0 (0.0)	24 (31.17)	<0.001	8 (9.76)	10 (4.08)	0.052	0 (0.00)	18 (15.38)	<0.001
Seizures, n (%)	8 (2.30)	0 (0.0)	8 (10.39)	<0.001	4 (4.88)	2 (0.82)	0.037	0 (0.00)	6 (5.1)	0.002
Ataxia, n (%)	6 (1.72)	5 (1.87)	1 (1.41)	0.792	2 (2.44)	4 (1.63)	0.638	3 (1.43)	3 (2.56)	0.671
Involuntary movements, n (%)	16 (4.58)	5 (1.87)	9 (11.69)	<0.001	4 (4.88)	9 (3.67)	0.628	3 (1.43(	10 (8.55)	0.003
Stroke / TIA symptoms, n (%)	35 (10.03)	0 (0.00)	35 (45.45)	0.003	14 (17.07)	18 (7.32)	0.010	0 (0.00)	32 (27.35)	<0.001
Anosmia, n (%)	73 (20.92)	68 (25.66)	5 (8.06)	0.013	6 (7.32)	67 (27.24)	<0.001	62 (29.52)	11 (9.40)	<0.001
Ageusia, n (%)	89 (25.50)	80 (30.19)	9 (14.52)	0.126	10 (12.20)	79 (32.11)	<0.001	72 (34.29)	17 (14.53)	<0.001
Muscle weakness, n (%)	160 (45.85)	129 (48.31)	30 (47.62)	0.921	19 (23.17)	138 (56.10)	<0.001	115 (54.76)	41 (35.04)	<0.001

Myalgia, n (%)	122 (34.96)	102 (38.49)	19 (30.65)	0.249	10 (12.20)	112 (45.53)	<0.001	96 (45.71)	25 (21.37)	<0.001
Paresthesia, n (%)	64 (18.34)	52 (19.62)	11 (17.74)	0.736	6 (7.32)	58 (23.58)	0.001	50 (23.81)	13 (11.11)	0.005
Diarrhea, n (%)	92 (26.36)	73 (27.24)	18 (23.68)	0.535	14 (17.07)	76 (30.89)	0.015	67 (31.90)	22 (18.80)	0.010
Increased sweating	115 (32.95)	102 (38.49)	12 (18.18)	0.002	7 (8.54)	108 (43.90)	<0.001	96 (45.71)	18 (15.38)	<0.001
Blood pressure <90/60 mmHg, n (%)	66 (18.91)	30 (11.19)	35 (45.45)	<0.001	13 (15.85)	40 (16.26)	0.931	29 (13.81)	24 (20.51)	0.115
Heart rate >100/min, n (%)	105 (30.09)	72 (26.87)	3 (40.26)	0.024	20 (24.39)	73 (29.67)	0.358	59 (28.10)	34 (29.06)	0.852
Hospital admission										
Oxygen therapy, n (%)<br>Not required<br>Nasal cannula<br>Non-re-breather mask<br>Non-invasive ventilation	 131 (37.53)<br>150 (42.50)<br>65 (18.62)<br>3 (0.86)	 119 (44.57)<br>117 (43.82)<br>28 (10.48)<br>3 (1.12)	 9 (11.69)<br>32 (41.56)<br>36 (46.75)<br>0 (0.00)	<0.001	 30 (37.04)<br>34 (41.98)<br>15 (18.51)<br>1 (1.23)	 98 (39.84)<br>110 (44.72)<br>34 (13.82)<br>2 (0.81)	0.849	 94 (44.76)<br>92 (43.81)<br>22 (10.48)<br>2 (0.95)	 34 (29.31)<br>51 (43.97)<br>30 (25.87)<br>1 (0.86)	0.003
MEWS score, n (%)<br>0-2<br>≥3	 330 (95.10)<br>17 (4.90)	 261 (97.39)<br>7 (2.61)	 65 (86.67)<br>10 (13.33)	<0.001	 78 (95.12)<br>4 (4.88)	 236 (96.33)<br>9 (3.67)	0.628	 204 (97.14)<br>6 (2.86)	 109 (93.97)<br>7 (6.03)	0.160
Laboratory tests										
Troponin I (mg/dL)	6.39 (3.24-16.41)	5.00 (2.72-11.88)	13.98 (5.80-33.47)	<0.001	10.18 (4.53-21.94)	5.46 (2.79-12.94)	0.001	5.00 (2.51-10.08)	10.45 (5.00-23.45)	<0.001
D-dimer (mg/L)	0.72 (0.44-1.48)	0.63 (0.41-1.23)	1.45 (0.72-3.23)	<0.001	1.00 (0.43-1.82)	0.66 (0.43-1.27)	0.042	0.61 (0.41-1.07)	1.01 (0.46-2.21)	<0.001

Values are presented as n (%), mean ± standard deviation, or median (interquartile range). COPD denotes chronić obstructive pulmonary disease; CNS, central nervous system; MEWS, Modified Early Warning Score; NSS, neurological symptoms or signs; TIA, transient ischemic

attack. High-risk NSS include decreased level of consciousness, delirium, seizures, stroke symptoms or transient ischemic attack. Low-risk NSS comprise headache, dizziness, decreased mood, and fatigue.

Table S2. Diagnostic accuracy for predicting in-hospital deaths or oxygen therapy

	AUC, 95% CI	Accuracy	Sensitivity	Specificity	PPV	NPV	LR
Diagnostic accuracy for the prediction of in-hospital mortality							
	AUC, 95% CI	Accuracy	Sensitivity	Specificity	PPV	NPV	LR
High-risk neurological symptom or sign	0.756 (0.659-0.853)	0.812	0.688	0.824	0.286	0.963	3.913
Low-risk neurological symptom or sign	0.697 (0.572-0.822)	0.765	0.619	0.775	0.159	0.967	2.754
High-risk / or absence of low-risk neurological symptoms or signs	0.741 (0.638-0.845)	0.682	0.810	0.673	0.145	0.981	2.477
Diagnostic accuracy for the prediction of in-hospital oxygen therapy							
	AUC, 95% CI	Accuracy	Sensitivity	Specificity	PPV	NPV	LR
High-risk neurological symptom or sign	0.630 (0.572-0.689)	0.555	0.322	0.938	0.896	0.457	5.24
Low-risk neurological symptom or sign	0.520 (0.457-0.584)	0.468	0.264	0.777	0.642	0.411	1.18
High-risk / or absence of low-risk neurological symptoms or signs	0.585 (0.522-0.645)	0.601	0.423	0.746	0.716	0.462	1.67

For abbreviations, see Tables 1-2. LR denotes likelihood ratio test; PPV, positive predictive value; NPV, negative predictive value.

Table S3. Multivariable regression analysis for death with stroke / TIA symptoms

	Univariable analysis		Multivariable analysis	
	OR (95% CI)	P-value	OR (95% CI)	P-value
Age, decades	2.08 (1.51-2.86)	<0.001	1.78 (1.18-2.69)	0.006
Prior CNS disease	8.13 (3.78-17.47)	<0.001	7.29 (2.72-19.59)	<0.001
Diabetes mellitus	2.33 (1.11-4.86)	0.025	-	-
Chronic kidney disease stage 3	4.52 (1.49-13.76)	0.008	-	-
Neoplasm	3.49 (1.47-8.08)	0.004	3.96 (1.32-11.83)	0.014
Stroke / TIA symptoms	5.06 (2.17-11.81)	<0.001	2.59 (0.78-8.65)	0.122
MEWS score (per point)	2.08 (1.51-2.88)	<0.001	2.25 (1.49-3.38)	<0.001
Troponin I (log)	1.60 (1.25-2.05)	0.002	-	-
D-dimer (log)	1.70 (1.26-2.28)	<0.001	-	-
AIC			133.65	
The Wald test, (df), P-value			34.98, (4), <0.001	
AUC (95% CI)			0.90 (0.86-0.94)	
The Hosmer–Lemeshow test P-value			0.924	

AIC denotes Akaike information criterion; AUC, the area under the receiver operating characteristic curve, CI, 95% confidence intervals; CNS, central nervous system; (df) degree of freedom; MEWS, Modified Early Warning Score; OR Odds Ratio, TIA, transient ischemic attack.

Table S4. Multivariable regression analysis for oxygen therapy with stroke / TIA symptoms

	Univariable analysis		Multivariable analysis	
	OR (95% CI)	P-value	OR (95% CI)	P-value
Age, decades	1.41 (1.2-1.62)	<0.001	1.34 (1.12-1.59)	0.001
Female sex	0.51 (0.33-0.79)	0.003	0.51 (0.29-0.89)	0.019
Prior CNS disease	2.56 (1.42-4.62)	0.002	-	-
Hypertension	1.96 (1.26-3.06)	0.003	-	-
Diabetes mellitus	1.69 (1.01-2.84)	0.048	-	-
Neoplasm	3.74 (1.52-9.18)	0.004	3.11 (1.07-9.00)	0.037
Stroke or TIA symptoms	3.21 (1.30-7.97)	0.012	2.09 (0.74-5.93)	0.164
MEWS score	5.01 (3.09-8,14)	<0.001	5.64 (3.17-10.07)	<0.001
Troponin I (log)	1.55 (1.26-1.92)	<0.001	-	-
AIC			311.73	
The Wald test, (df), P-value			49.29, (4), <0.001	
AUC (95% CI)			0.80 (0.75-0.85)	
The Hosmer–Lemeshow test P-value			0.077	

For abbreviations, see Table S3.

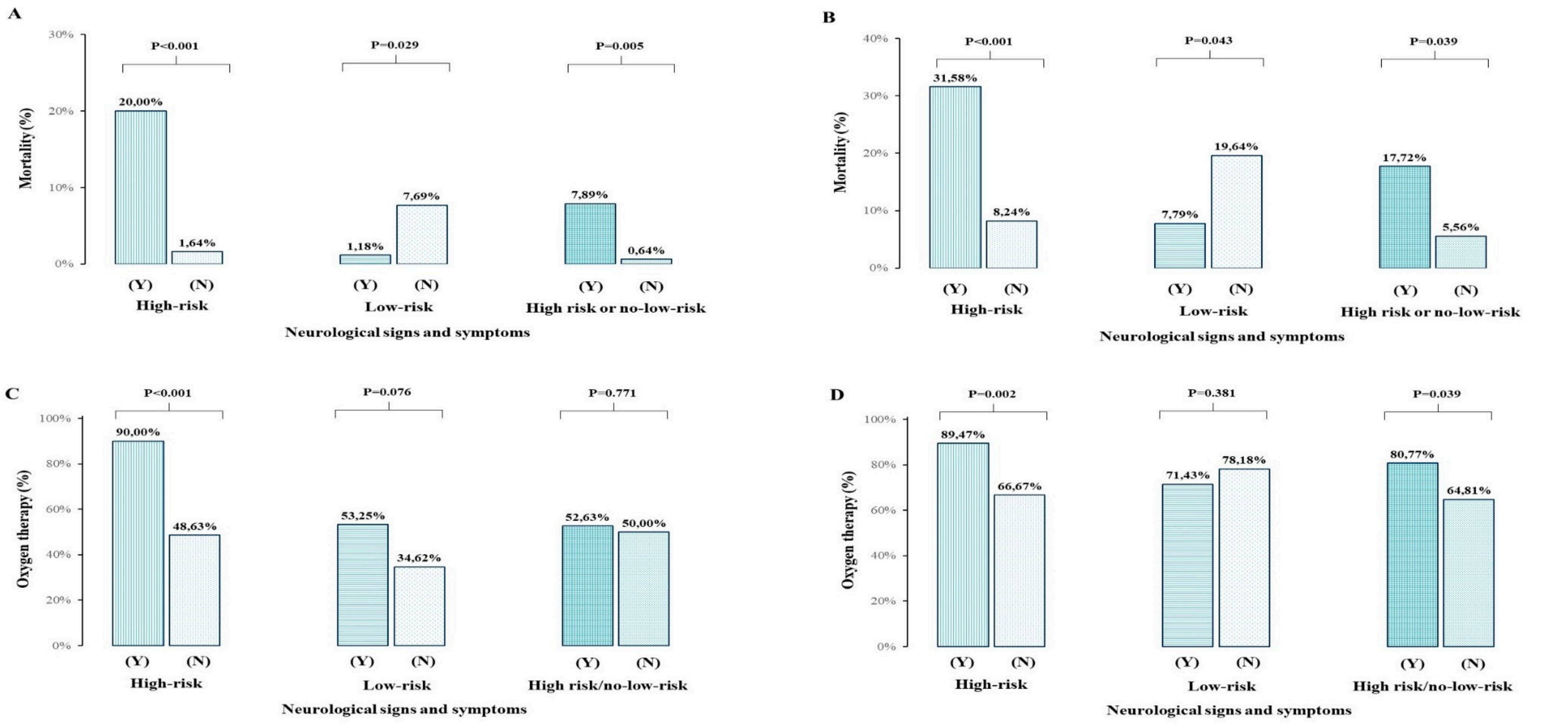

Figure S1. Mortality rates and requirement for oxygen therapy in COVID-19 patients according to the severity of neurological symptoms and signs and methods of data collection (A, C – prospective; B, D – retrospective).

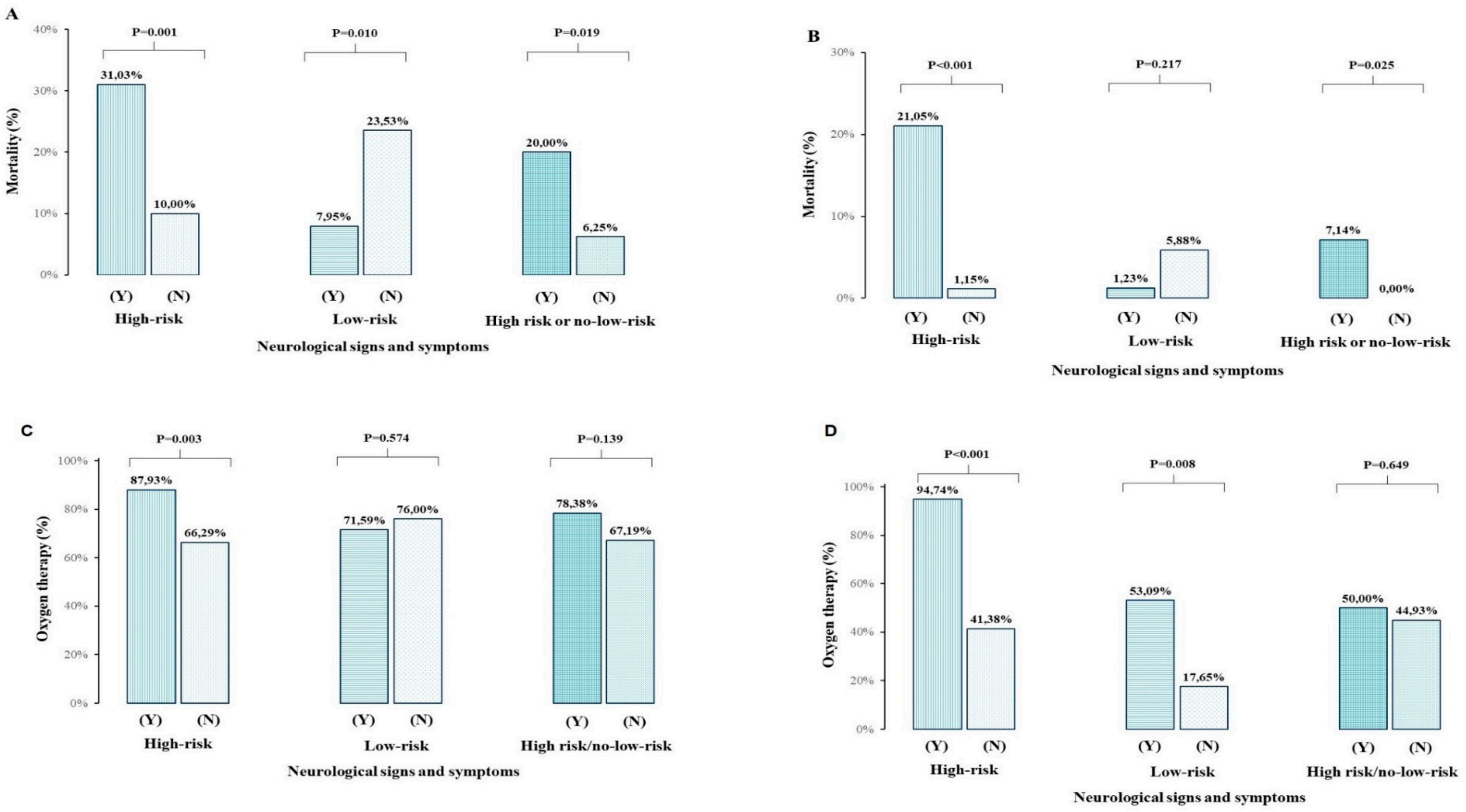

Figure S2. Mortality rates and requirement for oxygen therapy in COVID-19 patients according to the severity of neurological symptoms and signs (NSS) and hospital department (A, C – Neurology; B, D – Metabolic Diseases and Diabetology). As there were no deaths and high-risk

NSS in the departments of Internal Medicine, Otorhinolaryngology and Infectious Diseases, patients hospitalized at these departments were not taken into account during analysis.
